# Identification of Topping Responsive Proteins in Tobacco Roots

**DOI:** 10.3389/fpls.2016.00582

**Published:** 2016-04-28

**Authors:** Fei Li, Huizhen Zhang, Shaoxin Wang, Wanfu Xiao, Chao Ding, Weiqun Liu, Hongxiang Guo

**Affiliations:** ^1^College of Life Sciences, Henan Agricultural UniversityZhengzhou, China; ^2^College of Public Health, Zhengzhou UniversityZhengzhou, China

**Keywords:** tobacco, topping, roots proteome, physiology response, secondary growth, nicotine synthesis

## Abstract

The process of topping elicits many responses in the tobacco plant, including an increase in nicotine biosynthesis, and the secondary growth of roots. Some topping responsive miRNAs and genes have been identified in our previous study, but the mechanism of the tobacco response to topping has not yet been fully elucidated. In this study, topping responsive proteins isolated from tobacco roots were screened using two-dimensional electrophoresis. Of the proteins identified, calreticulin and auxin-responsive protein indole acetic acid (IAA9) were involved in the secondary growth of roots; leucine-rich repeat disease resistance, heat shock protein 70, and farnesyl pyrophosphate synthase 1 were involved in the wounding stress response; and F-box protein played an important role in promoting the ability of nicotine synthesis after topping. In addition, we identified five tobacco bHLH proteins (NtbHLH, NtMYC1a, NtMYC1b, NtMYC2a, and NtMYC2b) related to nicotine biosynthesis. NtMYC2 was suggested to be the main positive transcription factor, with NtbHLH protein being a negative regulator in the jasmonic acid (JA)-mediated activation of nicotine biosynthesis after topping. Tobacco topping activates a comprehensive range of biological processes involving the IAA and JA signaling pathways, and the identification of proteins involved in these processes will improve our understanding of the topping response.

## Introduction

Tobacco is an important economic crop, with the leaf representing the primary product. In order to improve leaf quality and production, the flowering head and young leaves of the tobacco plant are removed when the first flower of inflorescence appears. This cultivation technique for flue-cured tobacco is known as topping. Topping can switch the plant from its reproductive phase to its vegetative phase by altering a number of biological processes in the plant, leading to changes in nicotine biosynthesis, the hormonal balance, root development, and the source–sink relationship (Baldwin et al., 1994; Li et al., 2007).

Nicotine, a secondary metabolite of the tobacco stress response, is an important raw material for pharmaceuticals, bio-pesticides, and biochemical reagents. Therefore, nicotine not only plays a key role in the plant’s natural defense against herbivores and insects, but it is also a crucial substance contributing to the commercial value of the tobacco leaf. Nicotine is synthesized predominantly in tobacco roots, and then transported into leaves. Nicotine biosynthesis can be influenced by many factors, including developmental stages, hormones, and both biotic and abiotic stressors. The up-regulation of nicotine synthesis is a typical response of tobacco roots to topping. PMT, ODC, SAMS, and QPT are some key enzymes in nicotine synthesis. The activities of these enzymes are known to increase following tobacco topping, suggesting that topping can enhance the ability of roots to synthesize nicotine (Saunders and Bush, 1979). The expression of PMT, ODC, SAMS, and QPT increased in cell cultures of *Nicotiana tabacum* treated with MeJA, indicating that MeJA has a regulatory role in activating nicotine synthesis (Imanishi et al., 1998; Shoji et al., 2000; Winz and Baldwin, 2001). JA is a wounding responsive signaling molecule. JA levels increased 10-fold in damaged leaves within 90 min, and an increase was observed in the roots at 2 h after wounding (Baldwin et al., 1997).

Since tobacco topping is a form of mechanical wounding, it can induce JA synthesis in the wounded area. In addition, removal of the apical meristem reduces the source of IAA in tobacco aboveground parts after topping, which changes the distribution of IAA in tobacco plant. JA and IAA have been identified as two key phytohormones in the response of tobacco to topping (Zhang and Baldwin, 1997; Shoji et al., 2000; Shi et al., 2006). Furthermore, two bHLH transcriptional factors (NbbHLH1 and NbbHLH2) have been identified as positive regulators, and a third factor (NbbHLH3) as a negative regulator in the JA-mediated activation of nicotine synthesis (Todd et al., 2010). Therefore, multiple intersecting signal transduction pathways and diverse transcriptional regulatory factors are involved in the up-regulation of nicotine synthesis after topping (Xu and Timko, 2004). Further work is required, however, to fully elucidate the mechanisms involved in the regulation of nicotine synthesis by JA and IAA.

The secondary growth of roots is another important response to topping in the tobacco plant. It has been reported that the biomass, number, and activity of the roots increased significantly after topping (Zhang et al., 2006). Topping decreased the content of IAA in tobacco aboveground parts, which altered the source–sink relationship such that tobacco roots become an important growth center. However, the mechanism of secondary growth of tobacco roots induced by topping remains unclear.

Some previous studies have evaluated tobacco topping responsive genes. A set of 60 cDNAs was screened using subtractive hybridization of a phage library before and after topping. The differentially expressed genes identified were related to signal transduction pathways, transcription, translation, wounding stress, and alkaloid biosynthesis, including arginine decarboxylase (ADC), ODC, and SAMS (Wang et al., 2000). Furthermore, virus-induced gene silencing led to the identification of six transcription factors involved in the regulation of nicotine synthesis by JA: NbERF1, NbbHLH1, NbbHLH2, NbbHLH3, NbARF1, and NbHB1 (Todd et al., 2010). Genes and miRNAs showing differential expression levels before and after topping were analyzed by combining miRNA deep sequencing and suppression subtractive hybridization. This led to the proposition of a miRNA-mediated model for the topping response in flue-cured tobacco (Qi et al., 2012). Further, these authors demonstrated that NtNAC, a target transcription factor of miR164a, was involved in the auxin signaling pathway, and nicotine synthesis. Many studies investigating the tobacco topping response at a transcriptional level have thus been reported, the results of which make an important contribution to our understanding of the response of flue-cured tobacco to topping.

Topping results in a large and rapid change of JA and IAA content in the tobacco shoot, and a delayed response in the roots. Many molecular events at the protein level will certainly be successively involved in the response to topping. However, thus far, no studies have investigated the dynamic changes in the tobacco root proteome after topping. This study set out to screen topping responsive proteins in order to shed further light on the mechanisms involved in the response of tobacco plants to topping.

## Materials and Methods

### Plant Materials and Stress Treatments

Tobacco (*N. tabacum K326*) plants in the field were topped when the first flower of inflorescence came into bloom. Tobacco plants allowed to grow without topping were used as a control. The roots of the tobacco plants were removed from the soil 10 days after topping. The number of lateral roots was compared between topped and control tobacco plants. The roots in the field were also sampled at 0, 2, 6, and 24 h post-topping for proteomic analysis. These samples were immediately frozen in liquid nitrogen and then stored at –80°C until analyzed.

Tobacco plants in pots were randomly divided into groups to undergo seven different stress treatments. All stress treatments were performed at the point when the first flower of inflorescence bloomed. Tobacco plants were not topped in any stress treatments other than the topping stress treatment. (1) Topping treatment: the flowering head and young leaves were removed; roots were sampled at 24 h after topping. (2) 2,4-D treatment: leaves were sprayed with 50 μM 2,4-D solution; roots were sampled at 24 h after spraying. (3) MeJA treatment: leaves were sprayed with 100 μM MeJA solution; roots were sampled at 24 h after spraying. (4) Leaf damage treatment: leaves were punctured; roots were sampled at 24 h after treatment. (5) Cold treatment: plants were kept at 4°C for 48 h; roots were sampled at the end of the 48 h treatment period. (6) Drought treatment: plants were kept without water for 20 days; roots were sampled at the end of the 20-day treatment period. (7) Salt treatment: plants were treated with 200 mM NaCl solution for 15 days; roots were sampled at the end of the 15-day treatment period. All samples were immediately frozen in liquid nitrogen and then stored at –80°C until used for analysis.

### Extraction and Two-Dimensional Electrophoresis of Root Proteins

Tobacco roots (1 G) were ground into a fine powder in liquid nitrogen. Proteins were extracted with the trichloroacetic acid–acetone–phenol method (Guo et al., 2015). After 1000 μg of proteins were loaded into the immobilized strip (24 cm, pH 3–10), IEF was performed at 50 V for 12 h, 200 V for 3 h, 500 V for 1.5 h, 1,000 V for 0.5 h, and 10,000 V for 3 h with a Protean IEF Cell system (Bio-Rad). The strips were then equilibrated for 15 min with buffer I (50 mM Tris-HCl pH 8.8, 6 M urea, 4% SDS, 20% glycerol, and 0.1% dithiothreitol) and 15 min with buffer II (50 mM Tris-HCl pH 8.8, 6 M urea, 4% SDS, 20% glycerol, and 0.25% iodoacetamide). Protean Plus Dodeca Cell (Bio-Rad) was used to perform the second dimension of electrophoresis on 12.5% sodium dodecyl sulfate polyacrylamide gel. The electrophoresis was run at 50 V and 25°C until the samples deviated from the strips and then at 200 V until the bromophenol blue dye front reached the bottom of the gel. After staining with Coomassie brilliant blue R-250, the gels were scanned with ImageScanner II (Amersham Pharmacia) to obtain 2-DE image. The image was analyzed with PDQest8.0 software ^1^. Each spot was quantified as the accumulated intensity of the optical density in proportion to spot volume. Each protein sample was obtained from three independent tobacco plants, and the 2-DE analysis was performed in triplicate for each sample. Plants given no topping treatment were used as a control, and data were presented as fold change (increase or decrease) versus matched controls. The protein spots with a significant difference (*p* < 0.05) were considered to be “significantly differential proteins.” The significantly differential proteins were identified by 4800 Plus MALDI TOF/TOF^TM^ Analyzer (ABI). The default parameters of Mascot software (Matrix science Company) were used to perform sequence similarity searches against NCBInr Database [Viridiplantae (1093002) and EST_*Nicotiana* (412325)].

### Quantitative RT-PCR Analysis

Total RNA was isolated from tobacco roots using TRIzol reagent (Invitrogen, Carlsbad, CA, USA). The reverse transcription reactions were performed with One Step PrimeScript RT-PCR Kit (Perfect Real Time). TaKaRa SYBR Premix Ex Taq^TM^ II (Perfect Real Time) was used for quantitative real time PCR on a Bio-Rad IQ5 Real-Time PCR Detection System. The volume of the qRT-PCR reaction was 25 μL, and actin was used as the endogenous reference gene. Each reaction consisted of 2 μL of product from the diluted reverse transcription reaction, 12.5 μL of 2x SYBR Premix Ex Taq^TM^ II, 0.5 μL of primers (forward and reverse), and 9.5 μL of nuclease-free water. The reactions were incubated at 95°C for 30 s, followed by 40 cycles of 95°C for 5 s, 57°C for 30 s, and 72°C for 30 s. Three replicates were performed for each sample. Fold change was determined using the 2^–ΔΔ^*^C^*^t^ method and error bars represent the standard deviation (SD) of the mean. AVEDEV and Student’s *t*-tests were used to determine SD of the mean and significant differences between groups. The primer pairs used for quantitative RT-PCR are shown in Supplementary Table S1.

## Results

### Comparative Analysis of 2-DE Maps of Tobacco Root Proteome Following Topping

**FIGURE 1:**
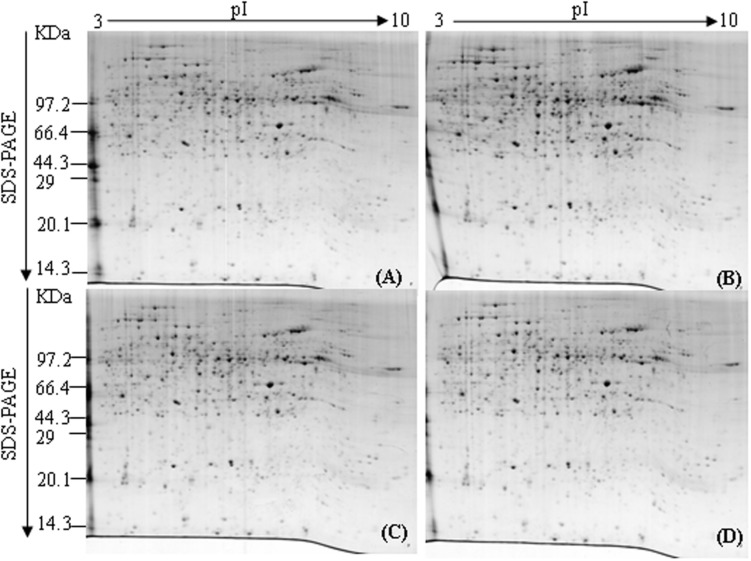
**2DE of tobacco roots proteome before and after topping. (A)** No topping; **(B)** 2 h after topping; **(C)** 6 h after topping; **(D)** 24 h after topping. 1000 μg of proteins were loaded, and the gels were stained with Coomassie brilliant blue R-250.

**FIGURE 2:**
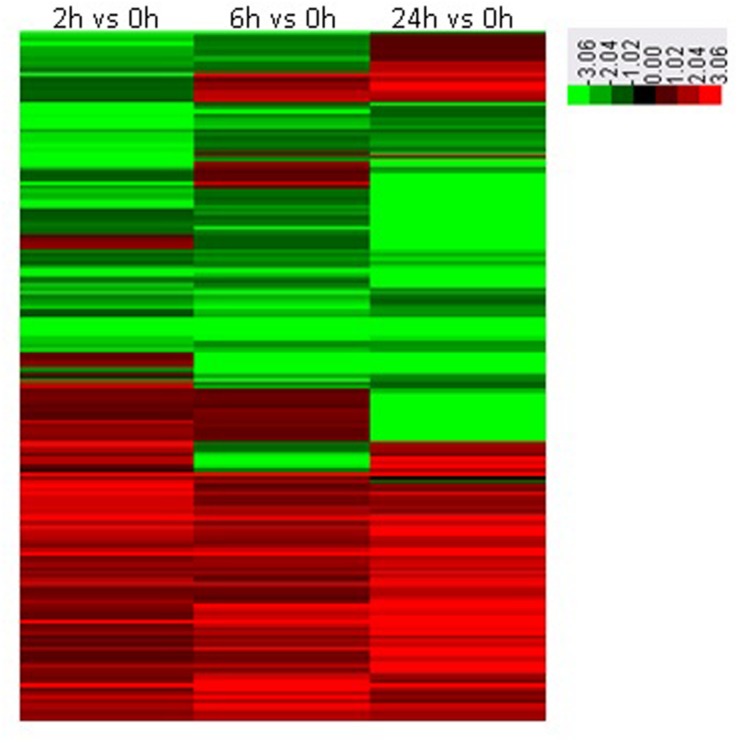
**Heat maps of significantly differential proteins following topping.** 0 h: No topping; 2 h: 2 h after topping; 6 h: 6 h after topping; 24 h: 24 h after topping. No topping treatment was used as the control.

To investigate the dynamic changes of tobacco roots proteome in response to topping, 2-DE analysis of the total proteins in roots before, and after topping was carried out. The 2-DE electrophoretic maps obtained from tobacco roots are shown in **Figure 1**. The protein spots showed a broad distribution in the pI range from 3.0 to 10.0 and the mass range from 10 to 120 kDa. In all, 184 significantly differential protein spots were detected between before and after topping (**Figure 2**).

### Identification of Differential Proteins in Tobacco Roots Following Topping

**FIGURE 3:**
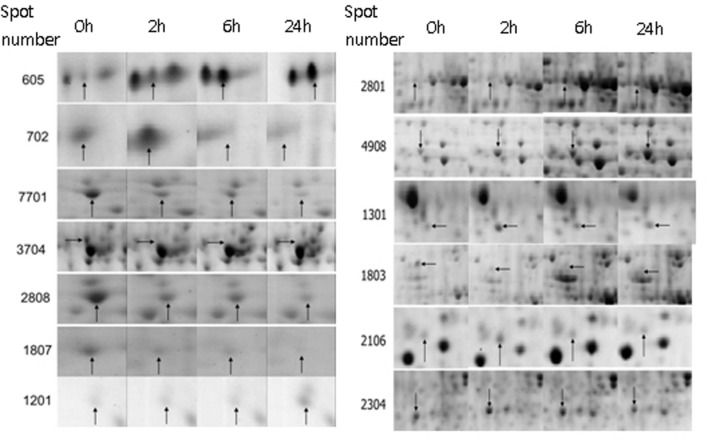
**The amplification map of differential proteins identified by MALDI-TOF-MS.** 0 h: No topping; 2 h: 2 h after topping; 6 h: 6 h after topping; 24 h: 24 h after topping. No topping treatment was used as the control. Spot number: the numerical order of differential proteins.

Thirteen significantly differential proteins were analyzed by MALDI-TOF-MS (Shown in **Figure 3**), and identified by searching the UniProt protein database (**Table 1**). These proteins included F-box protein, Leucine-rich repeat (LRR) disease resistance, CRT, Ras-related protein, bHLH, Heat shock protein 70 kDa (HSP70), Auxin-responsive protein IAA9, V-type proton ATPase subunit B, FPPS and proteinase inhibitor.

**Table 1:** The identification of differential proteins in tobacco roots following topping.

Spot number	Protein name	UniProt accession number	MW	pI	Fold change		
					2 h	6 h	24 h
2808	F-box protein	Q9SRD0	43.00	8.12	–3.85	–2.44	–7.14
1807	Proteinase inhibitor	P01078	6.03	7.68	–1.03	–1.52	–8.33
7701	LRR disease resistance	P84735	8.70	9.63	–3.85	–2.63	–5.88
1201	F-box protein	Q6NPQ1	43.65	9.59	+1.16	+1.51	+3.05
3704	Farnesyl pyrophosphate synthase 1	O24241	39.63	5.51	+1.13	+2.98	+3.19
605	Tobacco calreticulin	Q40401	47.68	4.45	+1.84	+3.25	+3.25
702	Tobacco calreticulin	Q40401	47.68	4.45	+2.15	–1.16	–1.27
1301	Ras-related protein	P28187	24.14	4.98	+2.67	+1.87	+1.94
1803	bHLH	A4D998	25.36	6.45	–1.15	–4.76	–3.85
2106	Auxin-responsive protein IAA9	AJ417876	28.43	7.57	+2.13	–1.28	–1.22
2304	Heat shock protein 70 kDa	P11143	70.87	5.22	+2.28	+2.35	+1.22
2801	V-type proton ATPase subunit B	Q40079	53.81	5.12	+1.24	+2.90	+2.62
4908	F-box protein	A4D998	25.36	6.45	+2.21	+3.16	+4.56

### Differential Proteins Related to Root Development Following Topping

**FIGURE 4:**
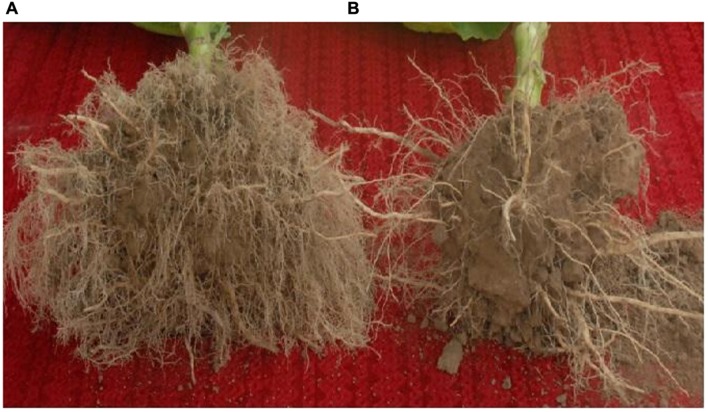
**The effect of topping on tobacco root development.** The roots of tobacco plants were taken out of the soil and photographed at 10 days after topping. **(A)** tobacco plant with topping; **(B)** tobacco plant without topping.

Plant root systems display considerable plasticity in response to endogenous and environmental signals. It has been previously shown that a change in root development is an important response of flue-cured tobacco to topping (Zhang et al., 2006). We found that the numbers of tobacco lateral roots markedly increased at 10 days after topping (**Figure 4**), indicating that growth of tobacco roots can be promoted by topping.

**FIGURE 5:**
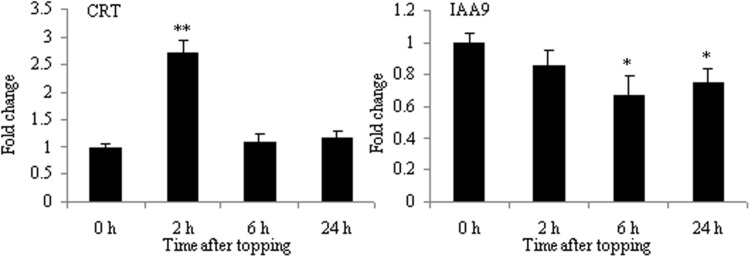
**RT-qPCR analyses of CRT and IAA9 in roots after tobacco topping.** CRT, calreticulin; IAA9, auxin-responsive protein IAA9. Fold change was determined using the 2^–ΔΔ*C*t^ method and error bars represent the standard deviation (SD) of the mean. Statistical significance: ^∗^*p* < 0.05 and ^∗∗^*p* < 0.01.

Calreticulin, an ER-localized Ca^2+^-binding protein, is a protein expressed differentially in the presence or absence of topping treatment. CRT plays a critical role not only in Ca^2+^ homeostasis but also in Ca^2+^ signaling mechanisms related to regeneration (Jin et al., 2005). Furthermore, it has been shown to modulate an array of responses to environmental stresses, such as cold and drought (Kim et al., 2013). As shown in **Figure 5**, the mRNA expression of CRT was up-regulated at 2 h after topping. This is consistent with the results of 2-DE, suggesting that CRT might play an important role in the growth of tobacco roots after topping.

The result of 2-DE demonstrated that IAA9 was down-regulated after topping (**Table 1**). Aux/IAA genes are involved in auxin-related root development processes, such as primary root elongation and lateral root formation (Fukaki et al., 2002; Tatematsu et al., 2004; Uehara et al., 2008). As shown in **Figure 5**, the mRNA expression of IAA9 was down-regulated after topping, suggesting that IAA9 plays an important role in the formation of lateral roots after topping.

### Differential Proteins Related to Damage Response Following Topping

**FIGURE 6:**
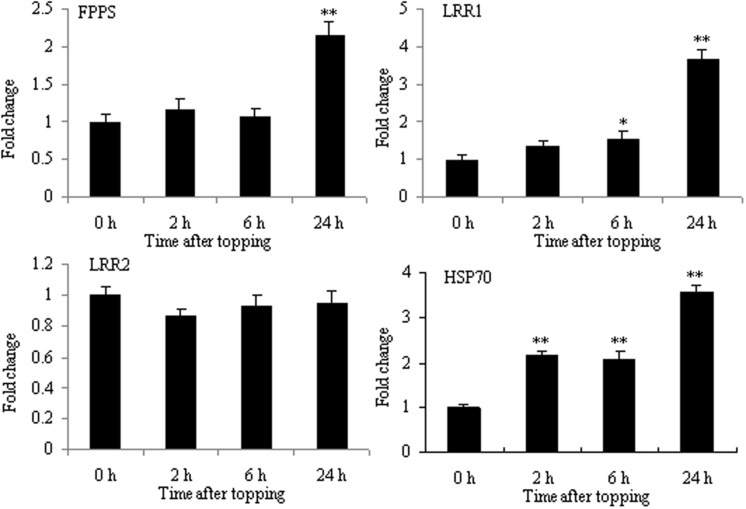
**RT-qPCR analyses of FPPS, HSP70, LRR1, and LRR2 in roots after tobacco topping.** FPPS, farnesyl pyrophosphate synthase; HSP70, heat shock protein 70; LRR, leucine-rich repeat protein. Fold change was determined using the 2^–ΔΔ*C*t^ method and error bars represent the SD of the mean. Statistical significance: ^∗^*p* < 0.05 and ^∗∗^*p* < 0.01.

Topping is a form of mechanical wounding. Plants have many molecules mediating a physiological response to wounding, such as JA and secondary metabolites. FPPS catalyzes the formation of farnesyl pyrophosphate (FPP) which is a key cellular intermediate in isoprenoid metabolic pathways. FPP is a biosynthetic precursor of steroid hormones, dolichols, and sesquiterpene phytoalexin. In addition, it has been reported that FPP has a role in signal transduction (Reilly et al., 2002). The proteomic analysis of tobacco roots showed that FPPS was markedly up-regulated after topping. As shown in **Figure 6**, the mRNA expression of FPPS was also up-regulated at 24 h after topping, suggesting that FPPS is involved in the response to topping damage. Hsp70 and LRR were the other two differential proteins. HSP70 is a stress responsive protein, and it was found to be up-regulated at the mRNA level after topping (**Figure 6**). In plants, the LRR disease resistance protein is the main class of disease resistance genes. The mRNA expression of LRR1 was markedly up-regulated at 24 h after topping, whereas the mRNA expression of LRR2 showed no obvious changes (**Figure 6**). Therefore, HSP70 and LRR1 are also implicated in the damage response following topping.

### Differential Proteins Related to the Regulation of Nicotine Biosynthesis Following Topping

**FIGURE 7:**
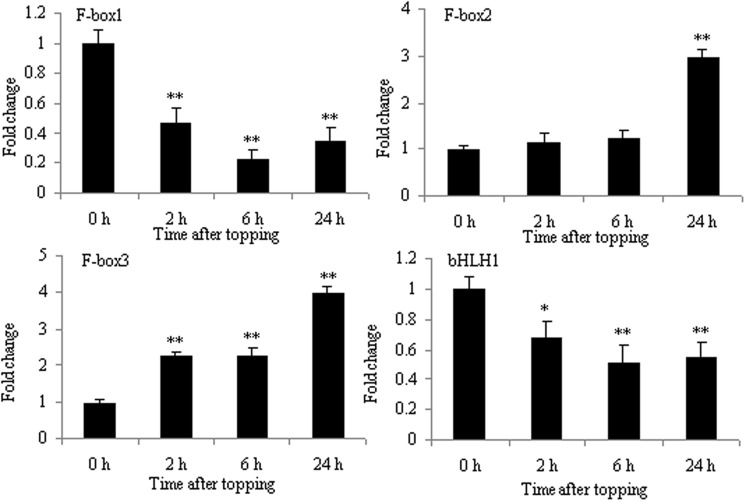
**RT-qPCR analysis of F-box1, F-box2, F-box3, and bHLH in roots after tobacco topping.** bHLH, basic helix-loop-helix. Fold change was determined using the 2^–ΔΔ*C*t^ method and error bars represent the SD of the mean. Statistical significance: ^∗^*p* < 0.05 and ^∗∗^*p* < 0.01.

The increase in nicotine biosynthesis is an important response of tobacco to topping. Tobacco F-box protein and basic helix-loop-helix protein (NtbHLH and nta000375) were two proteins expressed differentially before and after topping (**Table 1**). The protein family is the key component of ubiquitin ligase E3 in the SCF complex and is related to many biological processes, including hormone signal transduction, plant growth and development, and abiotic and biotic stresses. SCF^COI1^ can bind JA-Ile, thus initiating the degradation of jasmonate ZIM-domain protein (JAZ), which is a negative regulator of JA responses (Wang and Wu, 2013). The bHLH is a member of an important plant transcription factor family in response to environmental stresses. It has been reported that F-box protein and bHLH family proteins are involved in JA-mediated nicotine biosynthesis (Wang and Wu, 2013). NbbHLH1 and NbbHLH2 were positive regulators, whereas NbbHLH3 had negative regulatory function in the JA-inducible nicotine synthesis (Todd et al., 2010). Therefore, it is unsurprising that the mRNA expression of F-box2 and F-box3 proteins increased, and that the mRNA expression of F-box1 and NtbHLH (nta000375) protein decreased after topping (**Figure 7**).

### Several bHLH Proteins Related to Nicotine Biosynthesis

**FIGURE 8:**
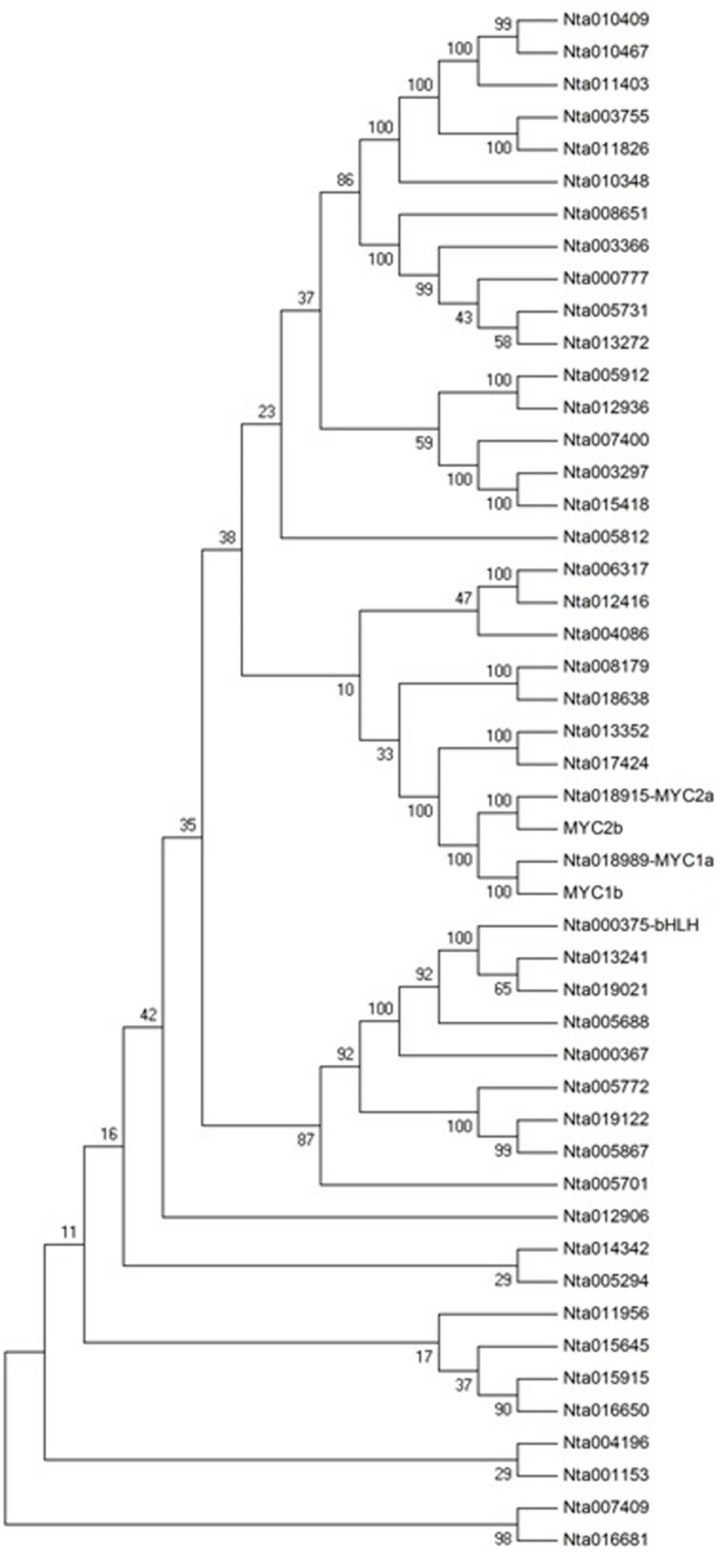
**Phylogenetic tree analysis of the bHLH transcription factor family in *Nicotiana tabacum*.** MEGA4.0 software and default parameters were used in the phylogenetic tree analysis. The 48 bHLH proteins are from TOBFAC databases in tobacco (http://compsysbio.achs.virginia.edu/tobfac/).

The bHLH family of proteins is a diverse group of functional transcription factors. There are 48 bHLH proteins from TOBFAC databases in the tobacco plant^2^. A phylogenetic tree of tobacco bHLH family proteins was constructed (**Figure 8**). NtMYC1a/1b and NtMYC2a/2b belong to MYC subfamily, and it has been shown that NtMYC2a and 2b are involved in regulating nicotine biosynthesis (Wang and Wu, 2013). NtbHLH (nta000375) protein does not belong to MYC subfamily, so it has a different role in regulating nicotine biosynthesis than do MYC subfamily proteins.

**FIGURE 9:**
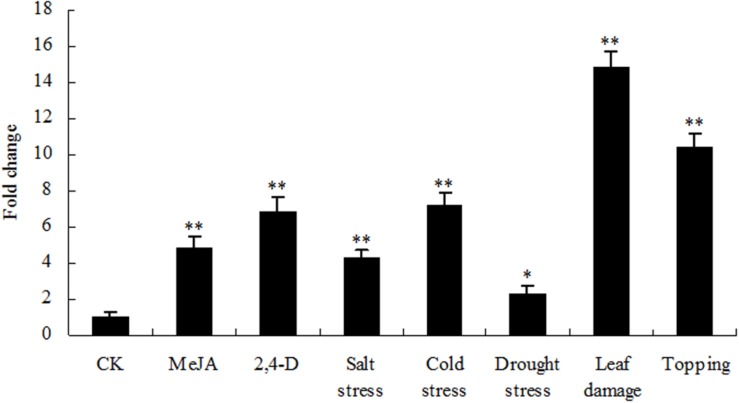
**RT-qPCR analysis of PMT in tobacco roots after stress treatment.** Fold change was determined using the 2^–ΔΔ*C*t^ method and error bars represent the SD of the mean. Statistical significance: ^∗^*p* < 0.05 and ^∗∗^*p* < 0.01.

**FIGURE 10:**
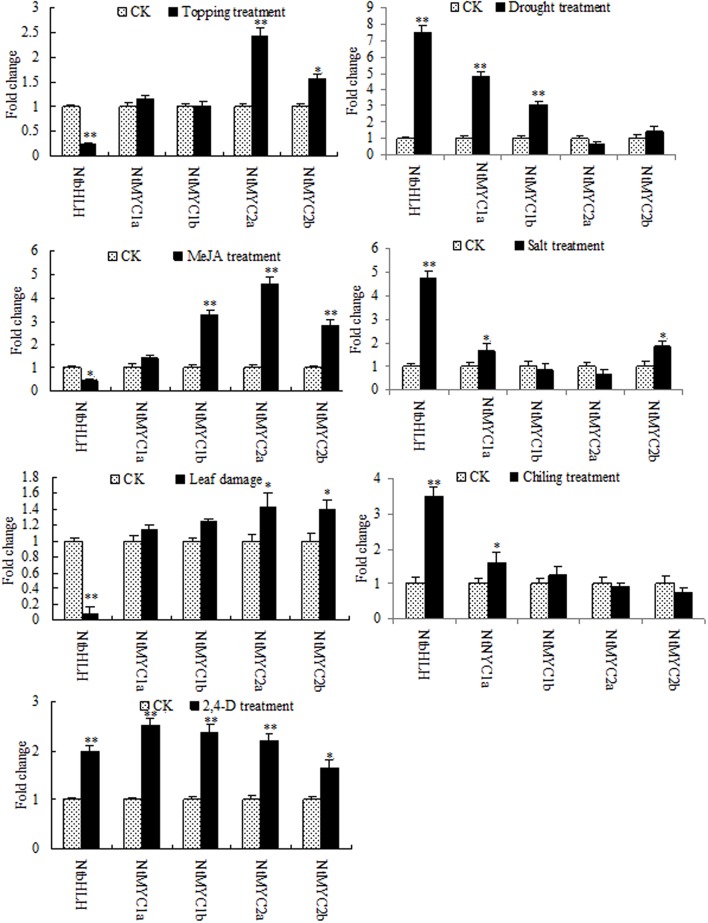
**RT-qPCR analyses of the five bHLH proteins in tobacco after stress treatment.** Fold change was determined using the 2^–ΔΔ*C*t^ method and error bars represent the SD of the mean. Statistical significance: ^∗^*p* < 0.05 and ^∗∗^*p* < 0.01.

Nicotine biosynthesis can be regulated by multiple biotic and abiotic stresses, such as drought stress, and herbivore or insect damage. PMT is the key enzyme in the nicotine biosynthetic pathway, and it regulates nicotine levels in tobacco. As shown in **Figure 9**, the mRNA expression of PMT can be increased by MeJA, 2,4-D, salt stress, cold stress, drought stress, leaf damage, and topping. NtbHLH protein was down-regulated by topping, leaf damage, and MeJA (**Figure 10**), indicating that NtbHLH protein is a negative regulator in the JA-mediated activation of nicotine synthesis. In addition, NtbHLH protein was up-regulated by 2,4-D, salt stress, cold stress, and drought stress, suggesting that it also has other functions in response to abiotic stress. The mRNA expression of NtMYC2a and NtMYC2b increased after MeJA, 2,4-D, leaf damage, and topping treatment, whereas NtMYC1a and NtMYC1b were markedly up-regulated by MeJA, 2,4-D, and drought treatment. Hence, there are different responses to biotic and abiotic stresses between NtMYC1a/1b and NtMYC2a/2b.

## Discussion

### Transformation of Growth Centre Promotes Secondary Growth of Roots after Topping

Topping is the removal of flowering head and young leaves (apical meristem) which is a primary source of auxin in the tobacco plant. Auxin (IAA) plays an important role in plant growth and development, such as plant cell elongation and division, the formation of the root hair, and the growth of the main roots and lateral roots. The primary auxin-responsive genes include three gene families: Aux/IAA (auxin/IAA), GH3 (Gretchen Hagen 3), and SAUR (Small auxin up RNA). Aux/IAA proteins possess a potent transcriptional co-repression activity and suppress the early induction of auxin-responsive genes. Topping reduces the synthesis of IAA in the aboveground part. This shifts the distribution of IAA in the tobacco plant, resulting in alterations in the expression of certain genes related to the auxin signaling pathway. In our previous studies, the expression of two auxin-related miRNAs, Nta-miRn49, and Nta-miR164a, was shown to be markedly repressed by topping (Guo et al., 2011). Axi 1, the target of nta-miRn49, plays a role in auxin activity (Richard et al., 1994). NAC1, the target of nta-miR164a, mediates auxin signaling to promote lateral root development (Xie et al., 2000). In the current study, the expression of IAA9 decreased after topping. These changes in expression of genes related to root development are helpful to our understanding of the secondary growth of roots after topping.

### Wounding Stimulus Is the Main Factor Promoting the Capacity of Nicotine Synthesis after Topping

Unlike animals, plants are unable to move in order to avoid environmental stresses, so they have instead evolved a comprehensive set of defenses to allow them to adapt to their environment. Topping is a form of damage sustained at the aboveground part, which triggers the wounding stress response. Nta-miR166c targets LRR family resistance proteins (McHale et al., 2006), and our previous work has demonstrated that nta-miR166c is significantly repressed by topping (Guo et al., 2011). The current study also found that several stress responsive proteins (LRR1, HSP70, and FPPS) were up-regulated after topping.

Plant jasmonates have important signaling roles in defense responses against abiotic and biotic stresses. Nicotine is a secondary metabolite produced by the defense responses. Coronatine-insensitive 1 (COI1), an F-box protein, is the key player in JA signaling. It has been demonstrated that the F-box protein is involved in jasmonate-inducible nicotine biosynthesis (Shoji and Hashimoto, 2011; Wang and Wu, 2013). COI1 is a part of an E3 ubiquitin ligase SCF^COI1^ complex, which is the receptor for the bioactive hormone JA-Ile. Most JA-induced responses are carried out by JA-Ile. JAZ proteins can repress JA signaling through interacting with MYC2, which is a positive transcription factor in JA-inducible nicotine biosynthesis. JA-Ile can promote the interaction between COI1 and JAZs, leading to degradation of JAZs and thus the activation of JA-inducible nicotine biosynthesis (Wang and Wu, 2013). The F-box protein is the target protein of nta-miR2111, expression of which was shown to be significantly up-regulated by topping in previous studies (Guo et al., 2011). The results of root proteomics in the current study showed that F-box protein was differentially expressed before and after topping, suggesting that it is involved in post-topping enhancement of nicotine synthesis.

In *Arabidopsis*, the MYC2-family bHLH transcription factors are involved in the regulation of jasmonate-responsive genes. The four tobacco bHLH proteins (NtMYC1a/b and NtMYC2a/b) belong to the *Arabidopsis* MYC2 clade. NtMYC1a/b and NtMYC2a/b are grouped together with NbbHLH1 and NbbHLH2, respectively, (Shoji and Hashimoto, 2011). NbbHLH1 and NbbHLH2 in *Nicotiana benthamiana* are positive regulators in the jasmonate activation of nicotine biosynthesis (Todd et al., 2010). It was found that NtMYC2 recognizes the G-box sequences in the promoter regions of several nicotine biosynthesis genes, including PMT2 and QPT2 (Shoji and Hashimoto, 2011). In the present study, the expression of PMT, NtMYC1, and NtMYC2 are clearly regulated by biotic and abiotic stresses, although the responses of NtMYC1 and NtMYC2 show some differences. As shown in **Figure 10**, NtMYC2 was up-regulated more than NtMYC1 after topping, whereas NtMYC1 was up-regulated more than NtMYC2 after drought treatment, suggesting that NtMYC2 is the main transcription factor involved in JA-mediated nicotine synthesis after topping. In addition to the four bHLH proteins, the results of proteomic analysis showed that NtbHLH (nta000375) protein was down-regulated after topping. NtbHLH does not belong to MYC subfamily, and it may be a negative regulator in the JA-mediating activation of nicotine synthesis.

## Conclusion

Topping of a tobacco plant triggers a comprehensive array of biological response processes, involving the transformation of the growth center and activation of the wounding stimulus response. IAA and JA signaling pathways play an important role in these responses.

## Author Contributions

The research was designed by WL. The experiments were performed by FL, HZ, SW, WX, and CD, and the data were analyzed by HG. The manuscript was written by HG.

## Conflict of Interest Statement

The authors declare that the research was conducted in the absence of any commercial or financial relationships that could be construed as a potential conflict of interest.

## Footnotes

1 www.bio-rad.com/products/pdquest/

2 http://compsysbio.achs.virginia.edu/tobfac/

## Abbreviations

bHLH: basic helix-loop-helix
CRT: calreticulin
FPPS: farnesyl pyrophosphate synthase 1
GH3: gretchen hagen 3
IAA: indole acetic acid
IEF: isoelectric focusing
JA: jasmonic acid
MeJA: methyl jasmonate
2,4-D: 2,4-dichlorophenoxyacetic acid
ODC: ornithine decarboxylase
PMT: putrescine *N*-methyltransferase
SAMS: *S*-adenosyl-L-methionine synthetase
SAUR: small auxin up RNA
QPT: quinolinic acid phosphoribosyl transferase.
