# Supplementary material for: Identification of Topping Responsive Proteins in Tobacco Roots
Source: Front Plant Sci. 2016 Apr 28;7:582. doi: 10.3389/fpls.2016.00582 (PMC4848317; doi:10.3389/fpls.2016.00582)
Supplement: Supplementary file 1 [file Table_1.DOCX]

Table S1 The primer for qPCR

| Name | Sequence | | GI number |
| --- | --- | --- | --- |
| Actin-F | GGAAACATCGTCCTTAGTGGTG | | gi\|50058114 |
| Actin-R | AATCCAGACACTGAACTTGCGT | |  |
| MYC1a-F | TGCAGGGGAGGAGAATAAGA | | gi\|296278605 |
| MYC1a-R | AGGAGTTCACCGACACATCC | |  |
| bHLH-F | GCAACTGATCGGAGATGGTT | | gi\|195970355 |
| bHLH-R | ATTCGAATCAGGGACAGCAC | |  |
| MYC1b-F | AGCAGAGAAATTGGCAGCTT | | gi\|296278607 |
| MYC1b-R | ACAAACCATCGTCTGAAGCC | |  |
| MYC2a-F | ACCAAGAGTCACTGCAACAG | | gi\|296278609 |
| MYC2a-R | CCGTGATAAAGTCAGGCGAA | |  |
| MYC2b-F | TTTGGGCCGGAACAAATACA | | gi\|296278611 |
| MYC2b-R | CTTTAATGGGTCTCCGGCAA | |  |
| F-box1-F | GATCCGGTACAGGACTACCA | gi\|190824662 | |
| F-box1-R | TGGCCCTAACTGTTTCCAAG |  |  |
| F-box2-F | TGCAATCAGTAGCAGCAGAG | gi\|192076287 | |
| F-box2-R | AATCCCTCCCTTTTGGATGC |  |  |
| F-box3-F | ACACCTTTGGTTAACGTCCC | gi\|191920565 | |
| F-box3-R | TCAGGGCAGATGATTTGAGC |  |  |
| LRR1-F | TAACGATTGGCCTGCTTTCT | | gi\|192198169 |
| LRR1-R | CCATCTCGTCACTGCCTTG | |  |
| LRR2 -F | ACGATTGCTGGTTCAAGTGT | | gi\|7672731 |
| LRR2 -R | CCATGTTACCAGCCTCAACA | |  |
| CRT-F | GGAGAGTTTCAACGATGGCT | | gi\|732892 |
| CRT-R | CCATTCCACTTGCCAGATGT | |  |
| FPPS-F | GCATGTGCACTTCTAATGGC | | gi\|255988558 |
| FPPS-R | TCTGGGTCAGCAAAACAGTC | |  |
| IAA9-F | AATGCCGGAGCAGGAATTAG | | gi\|16610192 |
| IAA9-R | CCCTTTCTCCGTTTCCTTTTAT | |  |
